# Circulating Epithelial Tumor Cells (CETC/CTC) in Prostate Cancer: Potential Prognostic Marker for the Risk of Recurrence During Radiotherapy

**DOI:** 10.3390/ijms26041548

**Published:** 2025-02-12

**Authors:** Dorothea Schott, Monika Pizon, Sonia Drozdz, Irina Mäurer, Georg Wurschi, Katharina Pachmann, Matthias Mäurer

**Affiliations:** 1 Transfusion Medicine Center Bayreuth, Kurpromenade 2, 95448 Bayreuth, Germany; mpizon@simfo.de (M.P.); kpachmann@laborpachmann.de (K.P.); 2 Department of Radiotherapy and Radiation Oncology, University Hospital Jena, Am Klinikum 1, 07747 Jena, Germany; sonia.drozdz@med.uni-jena.de (S.D.); matthias.maeurer@med.uni-jena.de (M.M.); 3 Department of Neurology, Neurooncology Center, University Hospital, Am Klinikum 1, 07747 Jena, Germany; irina.maeurer@med.uni-jena.de; 4 Clinician Scientist Program, Interdisciplinary Center for Clinical Research (IZKF), Jena Hospital, 07747 Jena, Germany; 5 Clinician Scientist Program OrganAge, Interdisciplinary Center for Clinical Research (IZKF), Jena Hospital, 07747 Jena, Germany

**Keywords:** prognostic biomarker, circulating epithelial tumor cells, prostate cancer, risk stratification, radiotherapy, PSA

## Abstract

Prostate cancer is a leading cause of cancer-related mortality in men, with radiotherapy (RT) playing a pivotal role in treatment. However, reliable biomarkers for assessing relapse risk following RT remain scarce. This study aimed to evaluate circulating epithelial tumor cells (CETC/CTC) as potential biomarkers for assessing relapse risk in prostate cancer patients undergoing RT. Peripheral blood samples were collected from 52 prostate cancer patients, and CETC/CTC were detected using the EpCAM surface marker. Patients received definitive, adjuvant, or salvage RT, and CETC/CTC counts were measured before, at mid-treatment, and at the end of RT. The association between changes in CETC/CTC counts and relapse risk was examined. CETC/CTC were detected in 96% of patients prior to RT. A significant reduction in CETC/CTC counts during RT, particularly in patients who had undergone surgery, was associated with a lower relapse risk. In contrast, an increase in CETC/CTC counts during or after RT was associated with a higher relapse risk (hazard ratio = 8.8; *p* = 0.002). Furthermore, 36% of patients receiving adjuvant RT and 14% of those receiving definitive RT relapsed, with a higher risk observed in patients showing increasing CETC/CTC counts during RT. Among patients receiving salvage RT, 18% relapsed, though changes in CETC/CTC counts were less significantly associated with relapse. Monitoring CETC/CTC levels during RT offers important prognostic insights into relapse risk in prostate cancer patients. While changes in CETC/CTC counts correlated with relapse, PSA levels measured during the study did not reliably reflect relapse risk in this cohort. CETC/CTC shows promise as a prognostic marker, though further studies are required to validate its clinical superiority over PSA.

## 1. Introduction

Prostate cancer is the most commonly diagnosed malignancy in men and the fifth leading cause of cancer-related death in men worldwide [1]. Radiotherapy (RT) is a cornerstone of treatment, either as a primary modality or in the postoperative setting with curative intent [2,3,4]. Randomized trials and meta-analyses have demonstrated the efficacy of radiation (either percutaneous therapy or brachytherapy) in reducing local recurrences at the highest level of evidence [5,6,7,8,9,10]. It is believed that prevention of local recurrences will also prevent subsequent distant seeding and reseeding from persistent reservoirs of locoregional disease. However, distant metastases can occur with or without prior local recurrence [11], highlighting the need for reliable markers to allow risk stratification and early detection of relapse [12]. Prostate-specific antigen PSA is routinely used as a tumor marker for prostate cancer patients [13]. After radical prostatectomy, PSA levels should fall below the detection limit, and any subsequent increase suggests recurrence. However, in definitive RT, PSA fluctuations can occur as a response treatment [14], making its interpretation less straightforward. Consequently, PSA alone is not sufficiently specific or precise for therapy monitoring [15].

To improve personalized diagnosis and individual therapy selection, molecular profiling has been explored in various solid cancers. Traditionally, DNA, RNA, or proteins from tumor biopsies have been analyzed [16], but this approach faces several limitations, including difficulty in obtaining metastatic tissue (especially in prostate cancer, where up to 90% of metastases occur in bone) [17], intra-patient and intra-tumor heterogeneity, and the impracticality of repeated tumor biopsies for monitoring tumor evolution.

Given these limitations, blood-based biomarkers—commonly referred to as liquid biopsies—have emerged as promising alternatives or adjunct to tissue biopsies and imaging studies [12] to better characterize tumor molecular drivers and response to treatment. In connection with the establishment of new multimodal therapeutic strategies for prostate cancer, the interest in biomarkers for therapy monitoring has increased [18].

**Figure 1:**
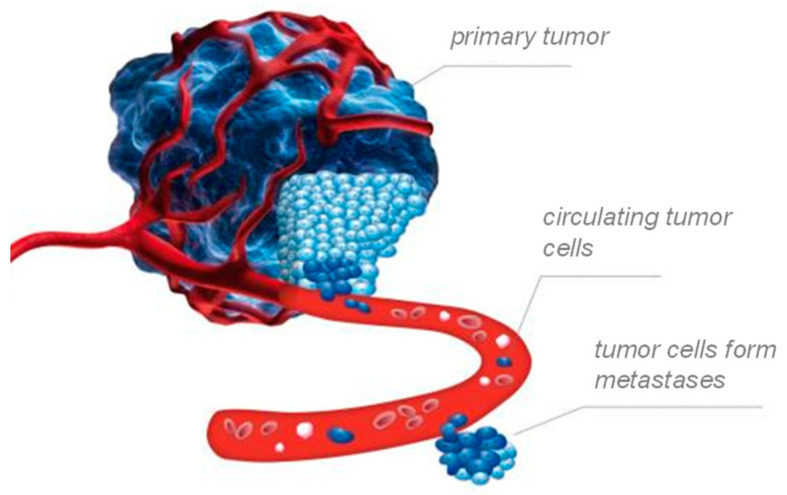
Schematic representation of the metastatic process.

Circulating epithelial tumor cells (CETC/CTC) known to be shed by the primary tumor into the bloodstream can settle in distant loci and have been considered responsible for the development of disease recurrence and metastasis (Figure 1). They preserve the heterogeneity of the primary tumor and reflect its characteristics [19]. CETC/CTC as prognostic and predictive biomarkers have been widely studied for various entities [20]. In particular, CETC/CTC appear to have prognostic significance in the context of radiotherapy [21,22]. Real-time biopsy, which is non-invasive and has the potential to monitor treatment response, detect minimal residual disease and manage non-invasive therapy resistance [23]. Detection of CETC/CTC by liquid biopsy could contribute to more targeted patient selection to identify those patients who will benefit from individualized, targeted therapy [24].

Recent studies have demonstrated that detecting circulating tumor cells (CTC) in the blood of metastatic prostate cancer patients allows for prognosis estimation [25].

We investigated CETC/CTC dynamics during definitive or postoperative radiotherapy in primary non-metastatic prostate cancer and assessed whether their behavior correlates with the risk of relapse (local recurrence, biochemical relapse, or metastasis) [26].

## 2. Results

For detecting cells of potential tumor-origin in peripheral blood, we used the EpCAM marker, expressed on epithelial cells but not hematological cells [22]. Enumeration of CETC/CTC followed standard blood cell counting methods, with direct staining minimizing cell loss [26].

Table 1 summarizes patient and disease characteristics. Peripheral blood samples from healthy volunteers (*n* = 15) served as negative controls, with no CETC/CTC detected.

The mean age of patients was 69.5 years, ranging from 53 to 82. 54% of patients were in stage III, 21% in stage II, 4% and 11% in stages I and IV, respectively. At the time of initial diagnosis, 78% of patients had no lymph node involvement. 36% had an aggressive tumor with a high risk based on the Gleason Score. Approximately 48% of patients underwent radical prostatectomy, with pathological diagnosis aligning with biopsy results. Before radiotherapy, 46% of patients received endocrine therapy.

The median follow-up period was seven years (range: 5–13 years). During this time, 10 events occurred, including five biological relapses, four distant relapses and one local recurrence.

### 2.1. Treatment Strategies and Outcome

**Figure 2:**
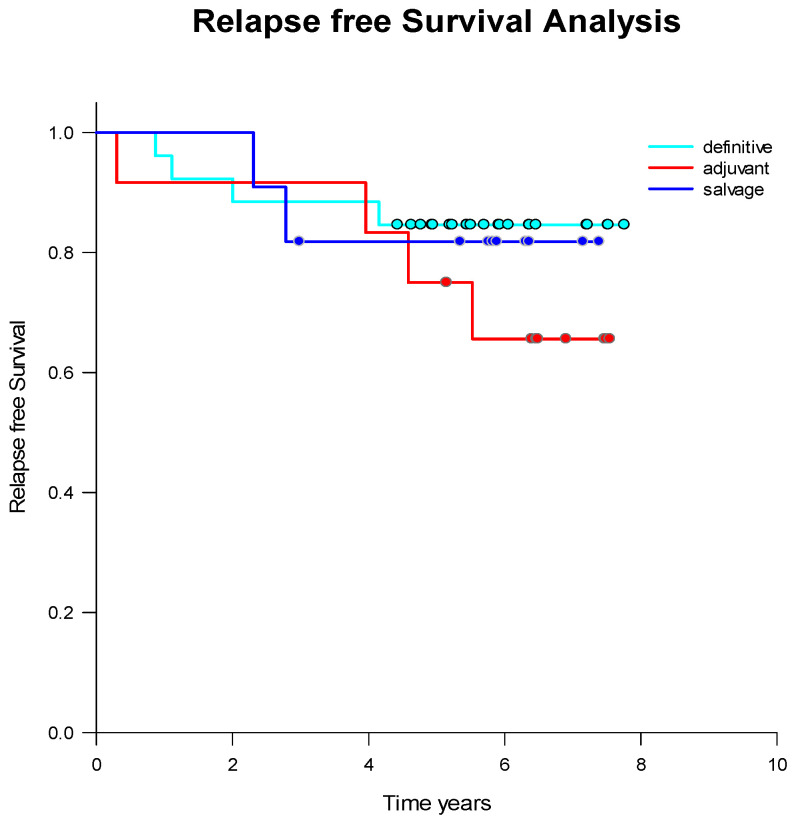
Kaplan-Meier estimates of PSA-relapse-free survival in prostate cancer patients treated with definitive, adjuvant, or salvage radiation. Dots represent censored patients.

Patients were treated based on age, risk factors, and personal preferences, following guideline recommendations. Treatment options included definitive radiotherapy without surgery, adjuvant radiotherapy after radical prostatectomy, or salvage radiotherapy in cases of biochemical relapse post-prostatectomy. Relapse-free survival did not differ significantly among these approaches, except for adjuvant radiotherapy, which showed a tendency toward poorer relapse-free survival (Table 2). However, this difference was not statistically significant (*p* = 0.05) (Figure 2).

Relapses occurred 2–4 years after treatment completion in both definitive and salvage treatment groups, whereas most relapses in patients treated with adjuvant radiotherapy occurred 4–6 years after treatment.

**Figure 3:**
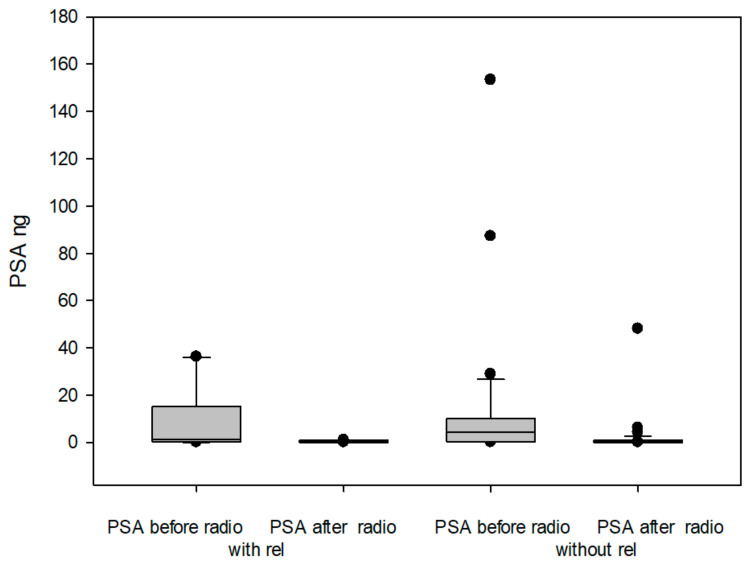
Boxplot diagram of PSA values before and after RT as a function of recurrence during the observation period.

Serum PSA levels significantly decreased during radiotherapy (median 3.2 ng/mL vs. 0.25 ng/mL; *p* < 0.001) (Figure 3). PSA was undetectable (<0.1 ng/mL) in 4% of patients prior to radiotherapy, while 35% of patients had undetectable PSA post-radiotherapy, indicating a positive therapy response.

However, there was no significant difference in PSA levels before or after radiotherapy between patients who later experienced relapse and those who remained in remission. PSA levels after radiotherapy were even lower in patients who experienced relapse. This suggests that PSA levels, either before or after radiotherapy, did not predict whether a relapse would occur.

### 2.2. CETC/CTC Numbers

#### 2.2.1. Before Radiotherapy, with and Without Surgery, and in Relation to Endocrine Therapy

Viable CETC/CTC were detected in 50 of 52 patients, either before or after RT (detection rate: 96%). Prior to RT, CETC/CTC were detected in 98% of patients (median 13 CETC/CTC/100 µL cell suspension). At midterm of RT, 82% of patients showed detectable CETC/CTC (median 8 CETC/CTC/100 µL cell suspension), and at the end of RT, 94% had detectable CETC/CTC (median 12 CETC/CTC/100 µL cell suspension). Tumor cell numbers ranged from 1 to 210 CETC/CTC per 100 µL of cell suspension. These findings suggest that tumor cells were released into the peripheral blood in almost all patients with early-stage prostate cancer.

**Figure 4:**
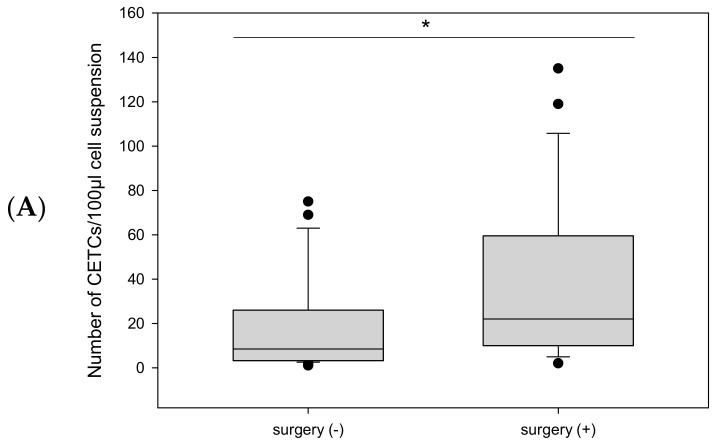

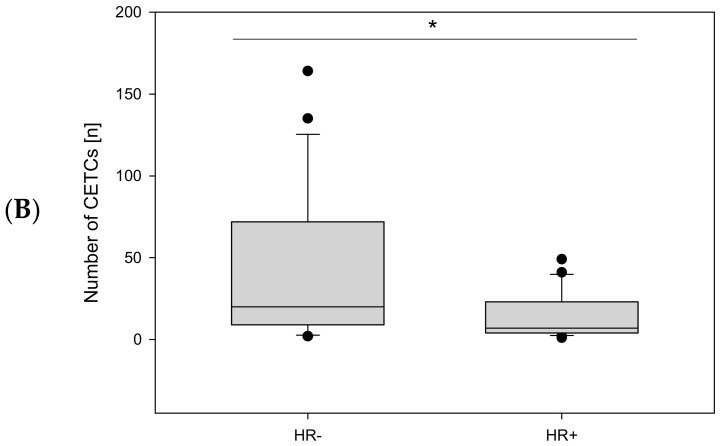
Boxplot diagram of CETC/CTC counts as a function of (**A**): surgical intervention or (**B**): androgen deprivation therapy. * corresponds to statistical significance.

Patients who underwent radical prostatectomy had significantly more CETC/CTC than those who did not undergo surgery (median 22 vs. median 8.5; *p* = 0.018) (Figure 4A).

Furthermore, patients who received endocrine therapy prior to radiotherapy had significantly fewer CETC/CTC compared to those who did not receive endocrine therapy (median 7 vs. median 20; *p* = 0.03) (Figure 4B).

#### 2.2.2. CETC/CTC Number During Radiotherapy

Although radiotherapy is applied locally to eliminate residual tumor cells after surgery or to treat patients who are ineligible for or unwilling to undergo surgery, changes in circulating tumor cell numbers were observed during treatment. The cell count trajectory was evaluated separately for different clinical scenarios (adjuvant, definitive, and salvage RT).

##### CETC/CTC Numbers in Patients Undergoing Adjuvant Radiotherapy

**Figure 5:**
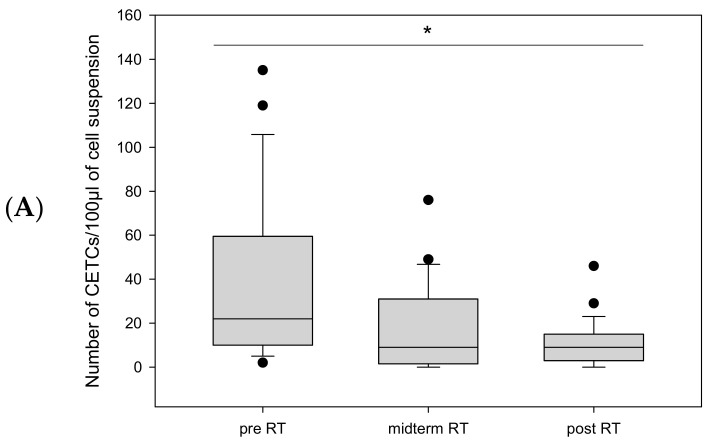

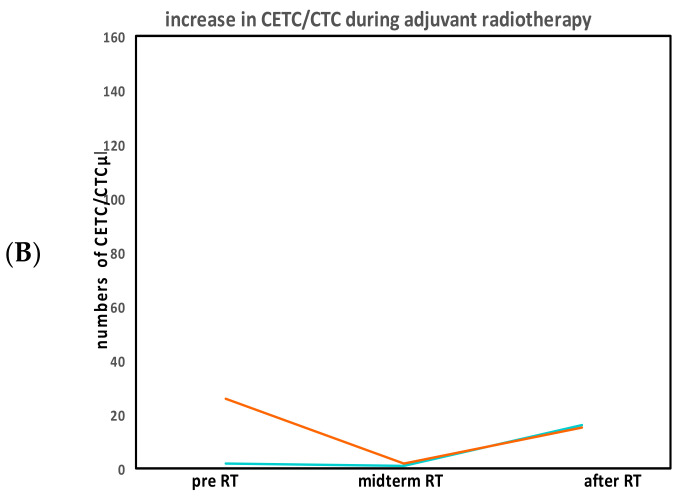
(**A**): Boxplot diagram of the mean number of CETC/CTC in patients after surgery during RT. (**B**): More than a twofold increase in CETC/CTC numbers was observed in 2 patients after surgery from midterm to the end of RT. * corresponds to statistical significance.

Twelve patients received radiotherapy after surgery due to adverse risk factors, with tumor cell counts available for 10 of them. These patients had the highest pre-radiotherapy CETC/CTC counts. During radiotherapy, CETC/CTC numbers decreased in 8 out of 10 (80%) (Median 22 vs. 9; *p* = 0.005) (Figure 5A), with the most pronounced decline occurring at midterm. However, in 2 out of 10 patients (20%), CETC/CTC counts increased more than two-fold from midterm to the end of radiotherapy (Figure 5B).

##### CETC/CTC Numbers in Patients Undergoing Definitive Radiotherapy

**Figure 6:**
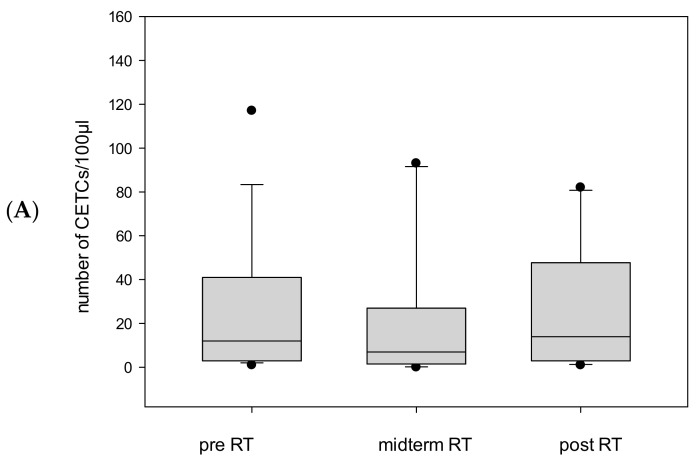

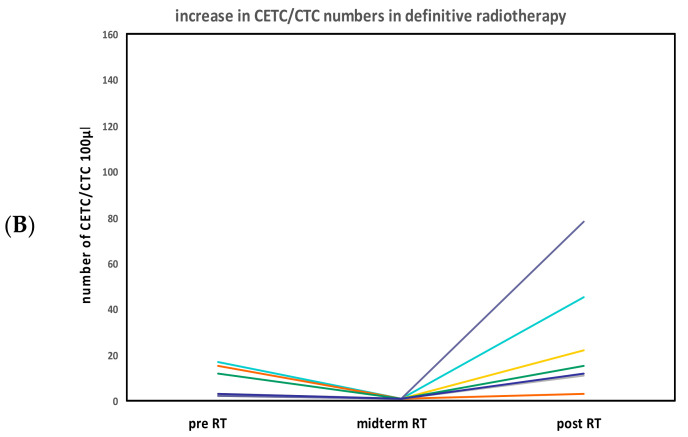
(**A**): Boxplot diagram showing the mean number of CETC/CTC in patients undergoing definitive RT. (**B**): More than a twofold increase in CETC/CTC numbers in eight patients receiving definitive radiotherapy from midterm to the end of RT.

The largest cohort in our study consisted of 29 patients who received definitive radiotherapy. Tumor cell counts were available for 27 of these patients. Unlike in the postoperative group, no similarly pronounced decrease in CETC/CTC numbers was observed in patients who did not undergo surgery (median 12 before vs. 13 after *p* = ns). By the end of radiotherapy, 19 out of 27 patients (70%) showed a reduction in tumor cell numbers (Figure 6A). However, in 8 out of 27 patients (30%); CETC/CTC counts increased from midterm to the end of radiotherapy (Figure 6B).

##### CETC/CTC Numbers in Patients Undergoing Salvage Radiotherapy

**Figure 7:**
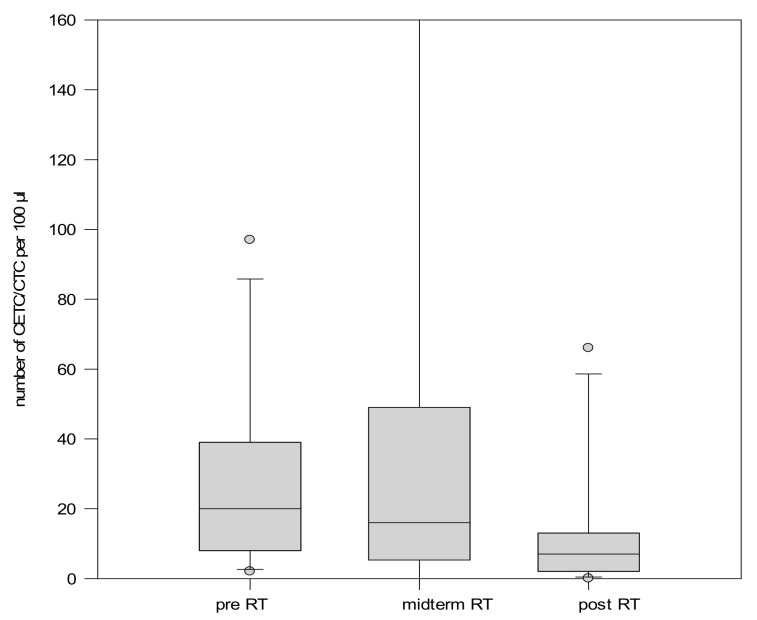
Boxplot diagram showing the mean number of CETC/CTC in patients undergoing salvage RT. The error bar for the midterm determination exceeds the scale. In order to keep the scales comparable, the scale was not expanded.

Among the 11 patients who received salvage radiotherapy, 10 exhibited a decrease in CETC/CTC numbers by the end of treatment, while only one showed an increase (Figure 7).

### 2.3. CETC/CTC Number Trajectory in Patients During Radiotherapy and Correlation with Relapse

Among patients who received adjuvant radiotherapy, 4 out of 12 (36%) experienced relapse within the following six years. Both patients with increasing tumor cells numbers from midterm to the end of radiotherapy (100%) relapsed, while only 1 out of 8 patients (12.5%) in the group with decreasing cell numbers relapsed. One additional patient, for whom cell count data was unavailable, also relapsed.

In the definitive radiotherapy group, relapses occurred in 4 out of 29 patients (14%). Three of the eight patients (37%) with re-increasing tumor cell counts after midterm relapsed, whereas only one patient (3.7%) with continuously decreasing tumor cell counts suffered relapse, which was a local recurrence.

Among the 11 patients who received salvage radiotherapy, two patients (18%) suffered relapse, both of whom were in the group with decreasing tumor cell numbers.

**Figure 8:**
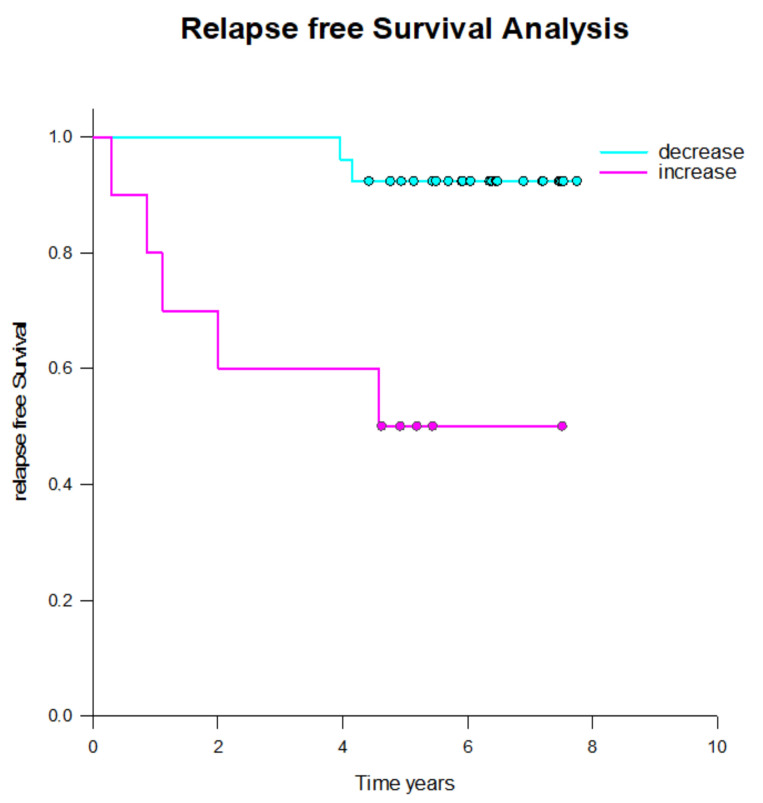
Kaplan-Meier survival curved for prostate cancer patients who received definitive or adjuvant radiotherapy, comparing those with decreasing (upper curve) and increasing (lower curve) CETC/CTC numbers (*p* = 0.002). Dots represent censored patients.

In patients treated for their primary tumor (both adjuvant and definitive radiotherapy), a significantly higher number of relapses occurred in those with increasing tumor cell counts by the end of radiotherapy compared to those with stable or decreasing circulating tumor cell numbers (Figure 8). However, this pattern was not observed in patients undergoing salvage radiotherapy. Salvage radiotherapy differs from both adjuvant and definitive radiotherapy, as the latter two are applied in a non-metastatic setting, whereas salvage therapy is generally considered palliative.

Notably, five patients with increasing tumor cell numbers by the end of radiotherapy did not experience relapse, suggesting additional parameters may play a role.

## 3. Discussion

Prostate cancer primarily affects men of higher age. Although complete surgical removal of the tumor seems to offer the most promising therapy to achieve cure, it is fraught with short-term but also long-term side effects that can significantly impact quality of life, particularly in frail patients. Additionally, some patients may opt against surgery due to the potential complications, the most common being impotence and urinary, or fecal incontinence.

As a result, radiotherapy has become an increasingly relevant treatment option for prostate cancer. It can be administered after surgery (adjuvant radiotherapy), as a primary treatment instead of surgery (definitive radiotherapy), or following a biochemical recurrence—defined as a rise in PSA levels after they had previously dropped below the detection limit following radical prostatectomy.

The primary goal of radiotherapy is to improve local tumor control and reduce the risk of local recurrence. In our patient cohort, there was no significant difference in relapse-free survival among the three treatment strategies, aligning with finding from previous studies [27,28]. Therefore, treatment decisions should be discussed with the patient.

A unique biomarker available in prostate cancer, PSA, is widely used for monitoring after radical prostatectomy. In the absence of remaining prostate tissue, PSA levels are expected to drop below the detection limit, and any subsequent increase is considered a biochemical relapse, even if no recurrence is yet visible on imaging.

This marker, however, has been shown to be controversial after radiotherapy [10,29]. While we observed a decrease in PSA levels during both adjuvant and definitive radiotherapy, there was no significant difference between patients who remained in complete remission and those who experienced relapse within the following six years. Thus, PSA levels did not serve as a reliable predictor of relapse.

This highlights an urgent need for more reliable biomarkers to assess recurrence risk in patients after radiotherapy. Such biomarkers would help identify patients at a higher risk of relapse, allowing for more personalized treatment strategies. Radiotherapy is administered with the expectation that it will eliminate prostate cancer cells—either as a standalone treatment, as an adjuvant therapy to target residual cells post-surgery to address regrowth following prior treatment. If metastases develop after radiotherapy, they must originate from cancer cells that either left the prostate site before treatment or survived radiotherapy and later spread to distant locations.

To investigate this, we examined whether circulating tumor cells could be detected in the peripheral blood during or after radiotherapy and whether their presence correlated with disease recurrence.

Circulating tumor cells are considered a source of distant relapse, which ultimately determines patient fate. While radiotherapy is generally thought to have only a local effect, we analyzed CETC/CTC numbers during radiotherapy across all three therapeutic strategies.

The maintrac^®^ approach used for detecting epithelial cells in peripheral blood does not apply any enrichment steps, which could reduce the yield of CETC/CTC. Instead, it identifies CETC/CTC among the white blood cells using an automated scanning fluorescence microscope, comparable to established blood-counting methods. This technique enables the detection of significantly more tumor cells compared to other methods [30], even in early-stages prostate cancer treatment.

Our findings indicate that radiotherapy does influence CETC/CTC numbers. Most patients experienced a decrease, which was most pronounced in those who had undergone prior surgery.

However, some patients—particularly in the adjuvant radiotherapy and definitive radiotherapy group—showed an increase in CETC/CTC numbers toward the end of treatment, often following a sharp decline at midtherapy. In contrast, during salvage radiotherapy, an increase in CETC/CTC was observed in only in one patient.

These finding suggest that radiotherapy not only exerts a local effect on remnant tumor cells in the prostate area but also has a systemic impact.

This aligns with the observation in breast cancer, where patients with increasing CETC/CTC numbers at the end of adjuvant radiotherapy had a significantly higher risk of relapse. In prostate cancer, 100% of patients with rising CETC/CTC after adjuvant radiotherapy relapsed compared to only 12.5% of those with decreasing CETC/CTC. Similarly, in the definitive radiotherapy group, relapses occurred in 37% of patients with increasing CETC/CTC, compared to just 3.7% of those with decreasing CETC/CTC. The hazard ratio for relapse in patients with increasing vs. decreasing CETC/CTC after radiotherapy for primary prostate cancer was 8.8 (*p* = 0.002).

These results demonstrate that the behavior of tumor cells in blood during radiotherapy—whether following surgery or as definitive radiotherapy—can predict later relapse. This suggests that CETC/CTC numbers could serve as a biomarker to identify patients at higher risk of recurrence, allowing for the development of additional treatments aimed at preventing relapse in prostate cancer [31].

However, it is important to emphasize that some patients with increasing CETC/CTC numbers have remained in complete remission. This indicates that additional important factors play a role in risk of recurrence. To avoid overtreatment in patients with rising CETC/CTC counts, we are currently analyzing additional biomarkers to better define the subset of patients who may require further intervention after radiotherapy.

## 4. Materials and Methods

### 4.1. Inclusion Criteria

The study included 52 patients with histologically confirmed prostate cancer who underwent definitive, adjuvant, or salvage RT at Jena University Hospital between April 2016 and March 2019.

Eligibility criteria were: male patients aged ≥18 years with histologically confirmed primary, non-metastatic invasive prostate cancer (stages I–IV), with any Gleason score and grading score. Prior chemotherapy or hormone therapy was permitted. Exclusion criteria for the study were the presence of distant metastases, prior malignancies within 10 years before prostate cancer diagnosis, or previous radiotherapy.

Blood samples were collected at three time points: before RT, midterm during RT, and on the last day of irradiation. Patients were followed up until 28 February 2023. The PSA value was determined as part of routine clinical checks before the start of treatment and after radiotherapy.

All patients were treated according to established guidelines. They received adjuvant RT with or without prior endocrine therapy, either before or after surgery.

Definitive radiotherapy was given according to the guidelines as normofractionated photon irradiation of the prostate with a total dose of 78.0 Gy (2.0 Gy per fraction). For the adjuvant setting or salvage irradiation, patients underwent a normofractionated total dose of 70.0 Gy (2.0 Gy per fraction). Patients who underwent irradiation to the regional pelvic lymph nodes received an additional 50.4 Gy on this anatomic site.

Statistical analysis was performed using SigmaPlot version 13.0 (Systat Software Inc., Chicago, IL, USA) for Windows. Comparisons between the variables were performed using Student’s *t* test (dichotomous variables) or ANOVA (for variables with more than two categories), with nonparametric tests applied where necessary. Correlations were analyzed using the Pearson or Spearman rank correlation coefficient. The Kaplan–Meier method, along with a log-rank test in SigmaPlot 13, was used to compare differences in relapse-free survival. A value of *p* < 0.05 was considered statistically significant.

The study was registered in the German Clinical Trials Register (DRKS00011840) and conducted in compliance with relevant regulations. Ethical approval was granted by the University of Jena on 13 September 2002, ethical Code 0921-08/02. All patients provided written informed consent.

### 4.2. CETC/CTC Analysis

Peripheral blood samples (7.5 mL) were drawn into EDTA tubes (EDTA was used as an anticoagulant) at three specified time points: Time point 1, on the first day of radiotherapy; Time point 2, midway through the radiotherapy series; Time point 3, on the last day of radiotherapy. Blood samples were transported from Jena University Hospital to the laboratory in Bayreuth within 48 h.

For CETC/CTC analysis, we used the maintrac^®^ approach [32]. 1 mL of blood is first taken from the EDTA tube and added to the red cell lysis. This is followed by a single centrifugation step. The remaining cell suspensions were then stained with a fluorescein-isothiocyanate (FITC)-conjugated anti-human epithelial cell adhesion molecule (Ep-CAMP) antibody (clone HEA-125, Miltenyi Biotec GmbH, Bergisch Gladbach, Germany) at a final concentration of up to 10^7^ cells/100 µL. After staining, the cells were transferred to wells of ELISA plates (Greiner Bio-one, Monroe, NC, USA), and propidium iodide (PI) was added to each sample to distinguish live from dead cells. Following one hour of sedimentation, immunofluorescence detection was performed using a Fluorescence Scanning Microscope (ScanR Olympus IX8 ZDC, Olympus, Hamburg, Germany). Each well was scanned by capturing 32 individual images, enabling both detection and relocation of cells for visual examination of EpCAM-positive cells. Data analysis was conducted using ScanR Analysis software (Olympus). Living EpCAM positive cells with intact morphology, but lacking nuclear PI staining, were classified as viable CETC/CTC and were the only cells counted for analyses. To ensure the consistency of sample analysis, fluorospheres (Fow-Check 770, Beckman Coulter, CA, USA) were used for daily verification of the microscope’s optical components and detectors.

### 4.3. Secondary Antibody Analysis

**Figure 9:**
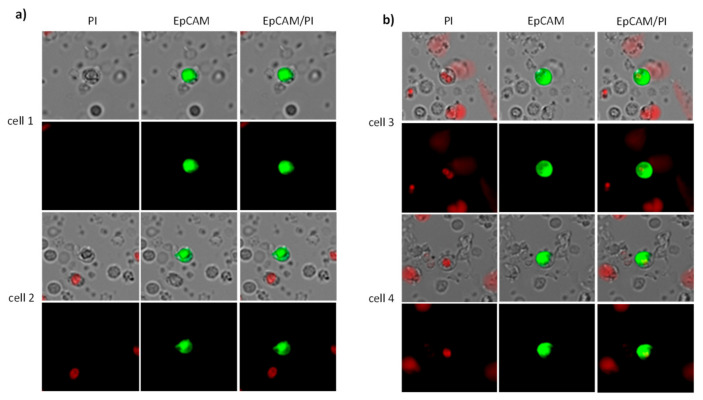
Circulating epithelial tumor cells. (**a**) Viable CETC/CTC: In viable cells, the cell membrane is well preserved, preventing the nuclear dye PI (red) from entering the cell. (**b**) Dead CETC/CTC: In apoptotic cells, the cell membrane becomes permeable, allowing the nucleus to be stained with PI.

The analysis of additional biomarkers on the CETC/CTC like PD-L1, PSA, and PSMA, was performed using an extended maintrac^®^ approach, as previously described [30]. Briefly, for PD-L1 analysis on CETC/CTC, we used an anti-human PD-L1 phycoerythrin (PE)-conjugated antibody (clone 29E.2A3, BioLegend, San Diego, CA, USA) at a final concentration of 0.2 µg/mL. For PSA analysis, an anti-human PSA PE-conjugated antibody (clone A67-B/E3, Santa Cruz Biotechnology, Dallas, TX, USA) was used, and for PSMA analysis, an anti-human PSMA PE-conjugated antibody (clone LNI-17, BioLegend, San Diego, CA, USA) was applied, both at a final concentration of 0.2 µg/mL. After staining, cells were visually inspected looking for green and red surface staining as well as a well-preserved nucleus (Figure 9). The results for staining with secondary antibodies were calculated as the percentage of the total number of CETC/CTC.

## 5. Conclusions

This study highlights the potential of circulating epithelial tumor cells (CETC/CTC) as a biomarker for predicting relapse in prostate cancer patients undergoing radiotherapy. While serum PSA levels were not a reliable predictor of recurrence after RT in this cohort, changes in CETC/CTC levels during treatment correlated with later disease recurrence. A significant decrease in CETC/CTC during RT, particularly in patients who underwent surgery, was associated with a lower risk of relapse. In contrast, an increase in CETC/CTC counts during or after RT was strongly linked to a higher risk of relapse. Although no clinical conclusions can be drawn due to the small number of cases, these results suggest that CETC/CTC monitoring may provide another method to identify patients at risk of relapse, enabling more tailored treatment strategies and potential interventions to prevent relapse.

## Figures and Tables

**Table 1:** Patient and disease characteristics.

	Patient Number (%)
Male	52 (100.0%)
Age	Mean 69.5 (min. 53–max. 82) years
Stage	
I	4 (8.00%)
II	28 (54.00%)
III	11 (21.00%)
IV	6 (11.00%)
n.a. ^1^	3 (6.00%)
T stage	
T1	8 (15.0%)
T2	24 (46.0%)
T3	15 (29.0%)
T4	1 (2.0%)
n.a.	4 (8.0%)
N stage	
negative	40 (78.0%)
positive	6 (11.0%)
n.a.	6 (11.0%)
Grading	
1	9 (17.0%)
2	17 (33.0%)
3	17 (33.0%)
n.a.	9 (17.0%)
Gleason Score	
2 + 3 = 5	1 (2.00%)
3 + 3 = 6	11 (21.00%)
3 + 4 = 7	13 (25.00%)
4 + 3 = 7	5 (10.00%)
3 + 5 = 8	3 (6.00%)
4 + 4 = 8	7 (13.00%)
4 + 5 = 9	7 (13.00%)
5 + 5 = 10	2 (4.00%)
n.a.	3 (6.00%)
RT-Treatment	
Definitive	29 (56%)
Adjuvant	12 (23%)
Salvage RT	11 (21%)
Risk groups	
Low risk	12 (23.00%)
Intermediate risk	18 (35.00%)
High risk	19 (36.00%)
n.a.	3 (6.00%)
PSA prior to RT	
<10 ng/mL	36 (69.2%)
>10 ng/mL	13 (25%)
n.a.	3 (5.8%)
PSA after RT	
<10 ng/mL	48 (92.3%)
>10 ng/mL	1 (1.9%)
n.a.	3 (5.8%)
Endocrine treatment	
Yes	24 (46.00%)
No	25 (48.00%)
n.a.	3 (6.00%)
Surgery	
Yes	25 (48.00%)
No	25 (48.00%)
n.a.	2 (4.00%)

^1^ not available.

**Table 2:** Frequency of recurrence in connection with the oncological setting.

	Adjuvant RT	Definitive RT	Salvage RT
No Relapse	8	25	9
Local recurrence (Prostate)	-	1	-
Biochemical recurrence	3	1	1
Distant metastasis	1	2	1

## Data Availability

The datasets generated and/or analyzed during the study are not publicly available due to preservation of privacy but are available from the corresponding author on reasonable request.

## Abbreviations

CETC/CTC	Circulating epithelial tumor cells
DFS	Disease-free survival
EpCAM	Epithelial cell adhesion molecule
FITC	Fluoroisothiocyanate
GAPDH	Glyceraldehyde 3-phosphate dehydrogenase
RT	Radiotherapy
